# Leaf habit and plant architecture integrate whole-plant economics and contextualize trait–climate associations within ecologically diverse genus *Rhododendron*

**DOI:** 10.1093/aobpla/plae005

**Published:** 2024-02-06

**Authors:** Juliana S Medeiros, Jean H Burns, Callie Dowrey, Fiona Duong, Sarah Speroff

**Affiliations:** Holden Arboretum, 9500 Sperry Rd, Kirtland, OH 44094, USA; Department of Biology, Case Western Reserve University, 10900 Euclid Ave., Cleveland, OH 44106USA; Holden Arboretum, 9500 Sperry Rd, Kirtland, OH 44094, USA; Holden Arboretum, 9500 Sperry Rd, Kirtland, OH 44094, USA; New England Aquarium, 1 Central Wharf, Boston, MA 02110USA

**Keywords:** anatomy, deciduous, evergreen, gas exchange, semi-evergreen, trait coordination

## Abstract

Plant resource strategies negotiate a trade-off between fast growth and stress resistance, characterized by specific leaf area (SLA). How SLA relates to leaf structure and function or plant climate associations remains open for debate, and leaf habit and plant architecture may alter the costs versus benefits of individual traits. We used phylogenetic canonical correspondence analysis and phylogenetic least squares to understand the relationship of anatomy and gas exchange to published data on root, wood, architectural and leaf economics traits and climate. Leaf anatomy was structured by leaf habit and carbon to nitrogen ratio was a better predictor of gas exchange than SLA. We found significant correspondence of leaf anatomy with branch architecture and wood traits, gas exchange corresponded with climate, while leaf economics corresponded with climate, architecture, wood and root traits. Species from the most seasonal climates had the highest trait–climate correspondence, and different aspects of economics and anatomy reflected leaf carbon uptake versus water use. Our study using phylogenetic comparative methods including plant architecture and leaf habit provides insight into the mechanism of whole-plant functional coordination and contextualizes individual traits in relation to climate, demonstrating the evolutionary and ecological relevance of trait–trait correlations within a genus with high biodiversity.

## Teaser

Leaves negotiate a trade-off between fast growth and stress resistance, but the timing and arrangement of plant parts may alter the costs and benefits of individual leaf traits within a given climate. Using phylogenetically informed analyses to investigate leaf traits related to carbon and water relations we found that correspondence between traits and climate was strongest in highly seasonal climates and that leaf carbon uptake versus water use are defined by different aspects of leaf morphology and anatomy. Our study shows that plant architecture and leaf habit contextualize individual traits in relation to climate, demonstrating the evolutionary and ecological relevance for suites of traits and mechanisms of whole-plant coordination within a genus with high biodiversity.

## Introduction

The leaf economics spectrum describes a trade-off between fast growth and stress resistance, whereby carbon investment per unit leaf area (specific leaf area, SLA) is thought to reflect leaf structural attributes like carbon allocation to palisade mesophyll and functional attributes like gas exchange rate and these together shape plant climate associations (Wright *et al.* 2004). Yet, the relationships between plant traits and environment are not straightforward, and progress on the question requires understanding how trait–environment relationships operate within groups of closely related species, and how individual traits integrate to affect a whole-plant phenotype (Anderegg 2023). If past selection has reduced variation in leaf economics traits, unexpected traits may drive the relationship between plant resource strategy and climate (Medeiros *et al.* 2017). Considering the correlations of leaf economics to leaf anatomy and gas exchange for closely related species can elucidate the dimensions of biodiversity on which natural selection can act, and help unravel the complexity of trait–climate associations by providing insight into how carbon is allocated among tissues which perform growth-related versus stress-resistance functions under different climate conditions. Moreover, species attributes like plant architecture, which integrates the functions of different plant organs, and leaf habit, which determines the timing of plant–climate interactions, could have over-riding effects that obscure or alter the fitness landscape of individual traits (Givnish 2002; Edwards *et al* 2014).

Genus *Rhododendron* is a great study system to address how different plant traits combine to determine resource use and allocation, and how trait combinations relate to plant–climate associations. Species within this genus are distributed across a wide latitudinal range from the sub-arctic tundra to the montane tropics, and they exhibit high diversity in leaf traits, including deciduous, evergreen and semi-evergreen leaf habits (Cox and Cox 1997). Deciduous *Rhododendron* species within section *Pentanthera* have higher SLA compared to evergreen species of section *Ponticum*, but within these two leaf habits a range of SLA is represented (Medeiros *et al.* 2017). Less well-understood, the semi-evergreen species within section *Tsutsusi* have intermediate SLA compared to deciduous and evergreen species, while their other traits can be more similar to evergreens, more similar to deciduous species, or they may have trait values that are outside the range exhibited by either deciduous or evergreen clades (Cai *et al.* 2014; Medeiros *et al.* 2020). The geographic range of semi-evergreen species also overlaps with that of deciduous and evergreen species, though the semi-evergreens tend to occur in more sunny locations and/or on volcanic soils characterized by seasonal water stress (Cox and Cox 1997), thus making them difficult to categorize in terms of resource acquisition strategy based on SLA alone.

One intriguing aspect of previous research on *Rhododendron* is that leaf carbon to nitrogen ratio (C:N) was more relevant than SLA for describing the relationships between leaves and wood traits (Medeiros *et al.* 2020). Although most studies of carbon economics do not include C:N, available evidence suggests that leaf habit in particular may have contrasting effects on SLA and nutrient stoichiometry, and leaf economics can interact with leaf habit to determine leaf resource acquisition strategy (Gorsuch *et al.* 2001; Zhou *et al.* 2017). Higher SLA is typical for deciduous species, and this can be driven by leaf anatomical differences such as less mesophyll, epidermis and vascular tissue, along with less airspace compared to evergreens (Poorter *et al.* 2009). Photosynthetic rates should theoretically be higher for species with proportionately thicker mesophyll, and mesophyll conductance is higher for species with greater air space fraction (Buckley *et al.* 2015), but the longer leaf lifespan of evergreen leaves leads to the expectation that they should exhibit lower gas exchange rates compared to deciduous species (Givnish 2002; Reich 2014). In fact, lower SLA can also be associated with thicker palisade and higher gas exchange rates (Schollert 2015; Gonzalez-Paleo and Ravetta 2018). Hence, the theoretical expectations for photosynthesis based on leaf anatomy, leaf economics and leaf habit are in contrast, which could lead to a disconnect between expectations for SLA versus C:N. Similarly, measures of leaf lifespan are often excluded from carbon economics studies, but the fact that deciduous and evergreen species can overlap in their SLA indicates that the two features could evolve independently. In addition, the ways in which carbon allocation, leaf anatomical structure and leaf habit interact to realize functions like gas exchange and shape trait climate associations is likely to be unique for clades with different evolutionary histories (Niinemets 2014; Yang *et al.* 2018) or different ecological associations (Wright *et al.* 2004). Consequently, the ways in which leaf anatomy and leaf economics combine to affect leaf resources acquisition strategy and subsequently how this impacts plant gas exchange under different climate conditions requires study of the timing of leaf surface area deployment in addition to their carbon allocation.

Not just leaves, but wood properties, roots traits and plant architecture also play important roles in the acquisition and allocation of resources across climate gradients, but the circumstances under which complex suites of traits might change in a coordinated fashion or independently of leaf resource acquisition strategy is not well-understood (Edwards *et al.* 2014). Even in cases where the relationship of SLA to leaf structure and function does not match with leaf economics theory, leaf anatomical traits can still be effective in explaining plant climate associations (Blackman *et al.* 2010), pointing to the idea that leaf economics traits need to be considered holistically within the context of other plant traits (Reich 2014). Studies within groups of closely related species show that changes in trait–trait relationships coincide with changes in habitat association and are important aspects of adaptive radiation (Males and Griffiths 2017; Sartori *et al.* 2019). For example, individual leaf traits of *Pelargonium* species do not correlate well with climate variables overall, but unique trait combinations are associated with particular habitats (Jones *et al.* 2013). Wood and root traits also contribute to plant resource acquisition, and higher SLA can be positively correlated with higher specific root length (SRL), or the carbon investment per unit root length (Withington *et al.* 2006). In contrast, for *Rhododendron*, SLA and SRL show different coordination when considering evergreen versus deciduous, and semi-evergreen clades, suggesting a critical role for leaf habit in their whole-plant resource acquisition (Medeiros *et al.* 2017). Furthermore, the idea that plant architecture (i.e. the size, number and spatial arrangement of plant parts) mediates relationships between plant organs has long been a part of trait coordination studies in plant hydraulics. To date, this topic has not been fully incorporated into studies of plant economics. For evergreen and semi-evergreen *Rhododendron*, larger xylem vessels in woody stems are associated with lower variation in SLA along the branch, showing that plant architecture interacts with leaf economics, potentially altering patterns of trait co-variation within plants, and the types of environments in which they can grow (Medeiros *et al.* 2020).

Thus, suites of traits define plant resource acquisition strategies, though SLA has been a fruitful framework relating leaf structure to functional attributes like plant climate associations (Wright *et al.* 2004; Poorter *et al.* 2009). More broadly, the evolution of new climate associations could be accompanied by changes in the allocation to any tissue which supports functions such as photosynthesis versus water conservation (Reich 2014). When considering suites of traits, our ability to understand how resource acquisition relates to climate may come down to accounting for shared evolutionary history, e.g. if strong past selection for low SLA reduced variation in SLA, this would be less likely to respond to subsequent climate warming, creating an apparent mismatch between SLA and current climate. For this reason, our understanding of how resource strategies relate to climate should be improved by looking at suites of traits within groups of closely related species, including traits like architecture and leaf habit which are shown to integrate plant function (Edwards *et al.* 2014) and which may moderate the climate experienced by actively growing plants (Givnish 2002).

In this study, we investigated leaf economics, leaf anatomy and leaf gas exchange rates for deciduous, semi-evergreen and evergreen *Rhododendron* species originating from different climates and growing at two botanical gardens. We compared leaf data to previously published data on root, wood and architectural traits for the same plants. We used phylogenetic comparative methods to address the following questions:

1) Do SLA, leaf life span and C:N co-vary among species within genus *Rhododendron* as expected based on the leaf economics spectrum?
2) Is there co-variation among leaf economics, anatomy and gas exchange, and does leaf habit influence trait–trait relationships?
3) What is the relationship of leaf habit, leaf economics, anatomy and gas exchange to root, wood and plant architectural traits?
4) Do leaf economics, anatomy and gas exchange differ according to species climate of origin?

## Materials and Methods

### Experimental design and plant growing conditions

Fully expanded current-year leaves were collected for examination of cross-sectional leaf anatomy in June 2014 and June 2015 at the Rhododendron Species Botanical Garden in Federal Way, WA (47.30°N, 122.31°W) and in May 2014 at the Holden Arboretum in Kirtland, OH (41.61°N, 81.30°W).

Gas exchange data were collected at the Holden Arboretum in 2013, 2014 and 2015. Leaf anatomy and gas exchange data collected for the current study were compared to data from published studies on the same plants examined here, including SLA and climate of origin estimates from Medeiros *et al.* (2017) along with leaf C:N, root morphology, wood anatomy and plant architecture from Medeiros *et al.* (2020). We examined 13 *Rhododendron* species that were present at both botanical garden study sites. Deciduous species included *R. arborescens*, *R. calendulaceum* and *R. molle*. Semi-evergreen species included *R. indicum*, *R. kiusianum*, *R. serpyllifolium* and *R. yedoense*. Lastly, evergreen species included *R. brachycarpum*, *R. catawbiense*, *R. hyperythrum*, *R. makinoi*, *R. maximum* and *R. smirnowii*. For all measures, three individual plants per species were examined at each botanical garden, and species mean values were computed for analysis. For three species examined at Holden Arboretum, only two individuals were sampled (*R. hyperythrum*, *R. maximum* and *R. serpyllifolium*).

The two gardens have a mean annual temperature of 10.5 °C and 8.8 °C and mean annual precipitation of 1034 and 1040 mm, respectively (Hijmans *et al.* 2005). At both gardens, *Rhododendron* are planted in locations that provide the light environment that species would experience in their native habitat, i.e. species native to sunny habitats are planted in sunny locations, while understory species are planted in the shade of coniferous and/or deciduous hardwood trees. Soil amendments added during planting differ across planting years and bed locations, so to avoid confounding species differences with possible differences in soil conditions across beds we used a stratified random sampling design: we intentionally chose bed locations that include plants representing more than one of the studied species, and for each species examined, we randomly chose three individuals located in different beds. The age-range of all *Rhododendron* plants examined was between 10 and 60 years old.

At Holden Arboretum, during the first year of establishment plants were provided with supplementary nutrients and water, but subsequently plants received only natural rainwater and were not provided with any fertilizer. Leaf litter inputs from overstory plants are removed annually with a leaf blower, and a 2–3” layer of hardwood mulch is applied as a top dressing approximately every 5 years. At the Rhododendron Species Botanical Garden plants are occasionally provided with supplementary water when severe drought occurs during the growing season, leaf litter inputs from overstory trees are not removed, and a 2–3” layer of mixed coniferous bark mulch is applied as a top dressing approximately every other year.

### Leaf cross-sectional anatomy

One upper-most fully expanded leaf from each plant was used for anatomical measurements, with a strip of tissue approximately 0.5–1 cm^2^ long that included one side of the leaf blade and the midrib being cut midway between the base and the tip of the leaf. Leaf pieces were embedded in paraffin and cross-sections 10 μm in thickness were made using a sliding microtome, and then mounted on a glass slide. The leaf sections were stained with a 1% Safranin O solution and viewed under a UV light microscope attached to a camera (Motic, Moticam 10, Hong Kong, China). Leaf blade characteristics were imaged at 100x magnification and midrib characteristics were imaged at 200× magnification. For leaf blade images, we intentionally avoided vascular bundles, such that the amount of mesophyll was maximized in the captured image. We used UV auto-florescence combined with image adjustment using software supplied with the camera to clearly distinguish different cell types in the leaf.

We used ImageJ (National Institutes of Health, Bethesda, MD, USA) to make anatomical measurements. For the leaf blade we measured the area occupied by three tissue types, epidermis (mm^2^, abaxial plus adaxial), palisade mesophyll and air space, as well as the total leaf area examined (mm^2^, length × width of section). The proportion of each tissue type per leaf area was calculated by dividing the area of each tissue type by the total leaf area examined. Leaf width was determined by drawing five separate lines perpendicular to the leaf blade, and the mean of those five numbers was taken to determine average width of the leaf section. We also determined mean midrib xylem conduit diameter by first measuring the area of individual midrib conduits and then calculating diameter based on the formula relating the area of a circle to its diameter. Diameters of all conduits in the midrib were averaged to obtain a single mean conduit diameter value for each plant. Finally, we determined total midrib xylem area by summing all the areas of the midrib xylem conduits.

#### Gas exchange

During June and July 2013, 2014 and 2015 we collected gas exchange data from plants growing at the Holden Arboretum using an open gas exchange system (model LI-6400, LiCor Biosciences Inc., Lincoln, NE). For each species, we measured one uppermost fully expanded leaf on the same three plants from which we collected anatomical samples, and from this we calculated species mean net assimilation or photosynthetic rate (*A*), transpiration (*E*), stomatal conductance (*g*_s_) and instantaneous water use efficiency (*A*/*E*; WUE). Cuvette conditions were as follows: reference CO_2_ = 400 ppm, block temperature = 20 °C, relative humidity = 45–55%, and photosynthetically active photon flux density = 1200 μmol m^−2^ s^−1^ provided using a red-blue light source (model LI-6400—02B, LiCor Biosciences Inc., Lincoln, NE) Leaves acclimated to cuvette conditions for a minimum of 10 min before recording a measurement. Upon reaching steady-state, we made six measurements over a 1-min period, and then these measurements were averaged to create a mean value for each plant.

#### Leaf economics

Published data on leaf economics (specific leaf area and carbon to nitrogen ratio) for the same plants examined in this study were obtained from Medeiros *et al.* (2017) and Medeiros *et al.* (2020). Leaf lifespan estimates for evergreen species were based on Cox and Cox (1997), while that for deciduous species was set at 0.5 years, since for *Rhododendron* species budbreak typically occurs around early May and leaf senescence occurs around late October. For semi-evergreen species, we estimated leaf lifespan for each species based on information presented in Cox and Cox (1997) combined with personal observation (J. Medeiros). *R. yedoense* and *R. serpyllifolium* were observed to maintain green leaves which over-winter, but these senesce during budburst the following year such that the entire canopy is replaced each spring, therefore we estimated leaf lifespan for these species to be 1 year. In contrast, *R. indicum* and *R. kiusianum* leaves were observed to over-winter and remain green and attached to the plant through the subsequent spring leaf flush, delaying senescence until the following fall, thus we estimated their leaf lifespan to be 1.5 years.

#### Climate of origin

To characterize species climate of origin, we used published data on species mean BioClim variables (Hijmans *et al.* 2005) retrieved for latitude and longitude points located within the native distribution of the species (Medeiros *et al.* 2017).

#### Species traits

We used published data for the same plants investigated in this study including root traits (first order root diameter, specific root length (SRL; Medeiros *et al.* 2017)), woody stem xylem vessel diameter (Medeiros *et al.* 2020) and plant architectural traits, including: mean size of leaves, leaf number per branch, total branch leaf area, Huber value and branch-level co-efficient of variation in specific leaf area (CVSLA; Medeiros *et al.* 2020).

#### Data analysis

Statistical tests were conducted in R (Version 4.2.1). All tests were conducted using phylogenetically informed analysis, and phylogenetic relationships were based on published data (Medeiros *et al.* 2017).

First, we conducted phylogenetic canonical correspondence analysis (CCA) using the *phyl.cca* command from the package ‘phytools’ (Revell 2012) to examine the correlations among leaf economics traits (i) species traits and (ii) climate of origin. In cases where the CCA indicated a significant effect of species traits or climate of origin, we further investigate the relationships of individual leaf economics traits to each of the species’ traits and climate variables, using PGLS in the package ‘caper’ (Orme 2013) to conduct backward stepwise model selection with a maximum likelihood estimate of lambda. For this, we set one of the leaf economics traits as the dependent variable (SLA, C:N or Leaf lifespan) and either all eight species traits or all eight climate variables were set as the independent variables. We then removed the independent variable with the lowest *t*-value in a stepwise fashion and chose the model with the highest adjusted *R*^2^.

Next, we investigated the relationships of leaf anatomy, leaf gas exchange and leaf economics, and we chose the predictors for these models based on the results of the CCA analysis of leaf economics, which showed a strong negative correlation of SLA and Leaf lifespan, combined with strong distinction of the three leaf habits across the first canonical axis (CA1; see Results ‘*Leaf economics in relation to species traits and climate of origin*’). We used PGLS to conduct two types of tests: (i) we tested for relationships of leaf anatomy to leaf economics using SLA, C:N and leaf habit as the predictors, with leaf anatomical features set as the dependent variable; (ii) we tested for relationships of leaf gas exchange to leaf economics, leaf habit and leaf anatomical traits. We only had gas exchange data for plants at Holden Arboretum, so only these data were included in the analysis, and gas exchange rates were set as the dependent variable. Predictors for the model included SLA, C:N, leaf habit and all five leaf anatomical traits including total epidermis area (TOTEP), proportion of palisade mesophyll (PAL), proportion of airspace (AIR), mean midrib conduit diameter (MRCD) and total midrib xylem area (MRXA).

Lastly, we investigated the potential relationships of leaf anatomy and gas exchange to species traits and climate of origin using CCA. For analyses of leaf anatomy, the total number of traits included in our study was larger than the number of species. Thus, we removed airspace proportion from the CCA analysis of leaf anatomy versus species traits based on the results of our PGLS analysis showing that airspace was strongly structured according to leaf habit. For the analysis of leaf anatomy versus climate of origin we removed temperature of the wettest quarter (*T*_wet_) based on the results of our CCA relating leaf economics to climate which showed that *T*_wet_ had low explanatory power and was strongly correlated with maximum temperature of the warmest quarter (*T*_max_). Following the CCA, to further investigate the relationships of individual leaf anatomical and gas exchange variables to each of the species’ traits and climate variables, we conducted backwards stepwise model selection using PGLS in the same manner as described above for leaf economics. For this, we ran models for all available variables, including those excluded from the CCA (i.e. airspace and *T*_wet_).

## Results

### Leaf economics in relation to species traits and climate of origin

**Figure 1.**
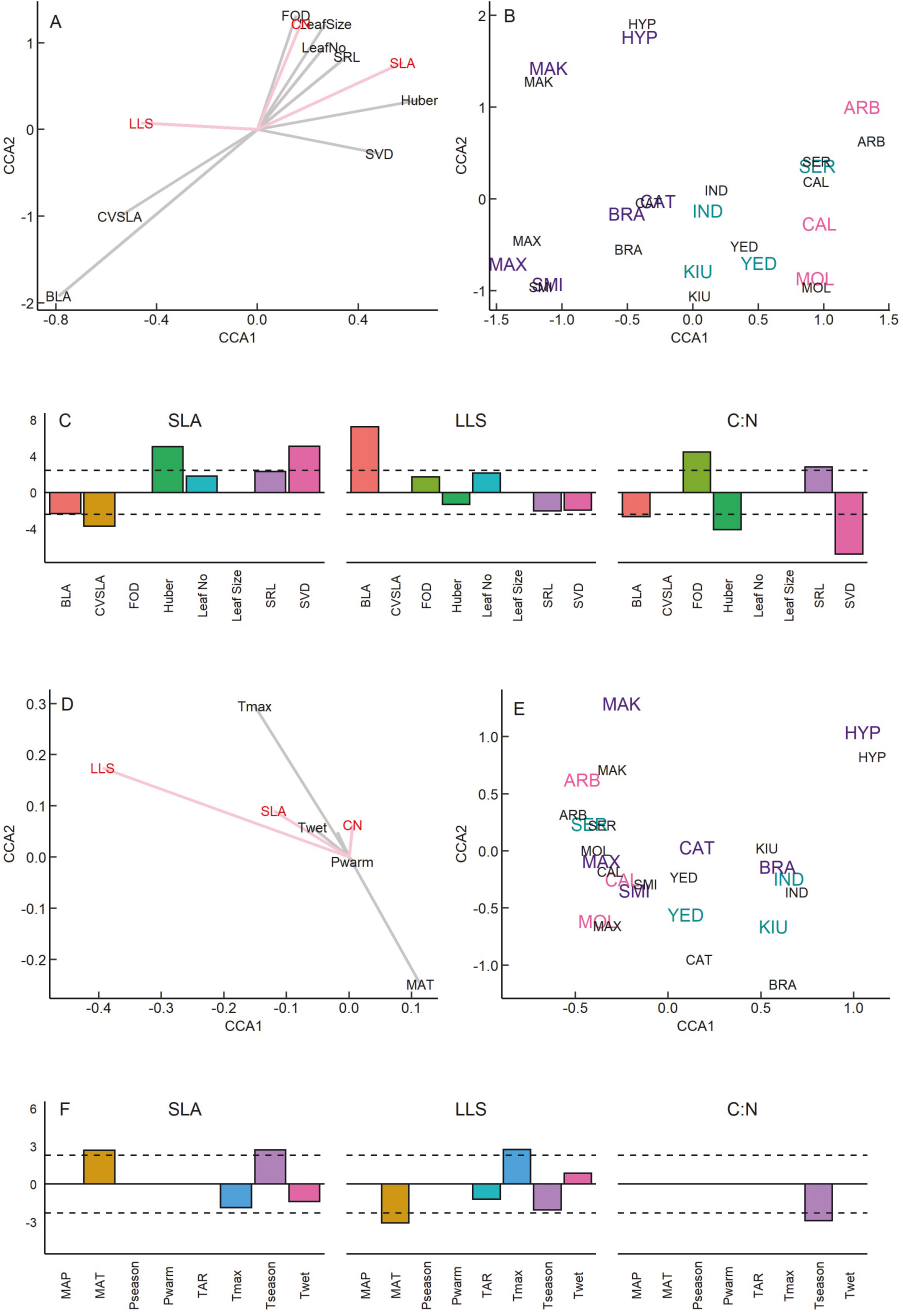
Canonical correspondence analysis (CCA) loadings, species scores and *t*-values from best-fit PGLS models investigating the relationships of leaf economics to other species traits (a–c) and species climate of origin (d–f) for 13 *Rhododendron* species representing deciduous, evergreen, and semi-evergreen leaf habits. For CCA loadings, red indicates traits in the leaf economic spectrum, grey indicates other species traits (a) or climate of origin (d). Species scores shown in small black font are for species traits (b) or climate of origin (e). Species scores for leaf economics are shown in large coloured font representing the different leaf habits and three letter codes indicate species names, purple = evergreen species (BRA, *R. brachycarpum*, CAT, *R. catawbiense*, HYP, *R. hyperythrum*, MAK, *R. makinoi*; MAX, *R. maximum*; SMI, *R. smirnowii*), teal = semi-evergreen species (IND, *R. indicum*; KIU, *R. kiusianum*; SER, *R. serpyllifolium*; YED, *R. yedoense*), pink = deciduous species (ARB, *R. arborescens*; CAL, *R. calendulaceum*; MOL, *R. molle*).

Phylogenetic canonical correspondence analysis (CCA) showed that leaf economics traits were significantly related to species traits along the first canonical axis (CCA1, Table 1). For leaf economics, CCA1 was composed primarily by SLA in opposition to Leaf lifespan, while CCA2 was composed primarily by C:N (Fig. 1A). For species traits, CCA1 was composed primarily by Huber value and stem vessel density (SVD) in opposition to branch leaf area (BLA) and the co-efficient of variation in SLA (CVSLA), while CCA2 was composed primarily by first order root diameter (FOD), leaf size, leaf number and specific root length (SRL). Species scores for both leaf economics and species traits were structured according to leaf type across CCA1, and across this axis the correspondence between leaf economics and species trait scores was very high (Fig. 1B). Across CCA2, a few species showed very strong correspondence between leaf economics and species trait scores (SER, CAT, SMI and MOL), while for other species their leaf economics score was either higher or lower compared to their species trait score. The best fit PGLS models showed that CVSLA, Huber value and SVD were all significant predictors of SLA, while BLA was the only significant predictor of leaf lifespan (Supplementary Information—Table S1; Fig. 1C). While C:N did not contribute substantially to CCA1, our best-fit models showed that it was significantly related to species traits BLA, FOD, Huber value, SRL and SVD.

**Table 1.** Chi-square values from phylogenetic canonical correspondence analysis testing for relationships of leaf economics, leaf anatomy and leaf gas exchange to climate of origin and other species traits (root, wood and architectural traits) for 13 *Rhododendron* species representing deciduous, evergreen and semi-evergreen leaf habits. Only the first two axes are shown for each analysis. Bold indicates significance.

X matrix	Y matrix	CC1	CC2
Leaf economics	Species traits	50.69**	20.22
	Climate	40.95*	7.91
Leaf anatomy	Species traits^††^	50.83*	24.14
	Climate^†^	25.32	8.69
Gas exchange	Species traits	40.81	23.44
	Climate	53.53**	25.34

^†^Denotes factors excluded from the analysis; ^†^*T*_wet_, ^††^airspace proportion.

*P < 0.05, **P < 0.01.

Leaf economics also showed a significant relationship with climate variables along CCA1 (Table 1). For leaf economics, CCA1 was composed primarily by leaf lifespan with a smaller contribution by SLA (Fig. 1D), and CCA2 was composed primarily by C:N. For climate variables, CCA1 was composed primarily by mean annual temperature (MAT) and maximum temperature of the warmest quarter (*T*_max_), while CCA2 was composed of variables that contributed little to the variance in the data. Species scores for both leaf economics and climate variables were structured according to climate of origin (Fig. 1E). Along CCA1 species scores for leaf economics and climate showed similar correspondence, with those from more seasonally variable climates clustered together on the left-hand side of the axis (MAK, ARB, SER, MOL, MAX, CAL and SMI) while species from climates with less variation in temperature were dispersed across the central and right side. Across CCA2 correspondence between leaf economics and climate scores was higher for some species (SER, CAL, SMI and IND) while the remaining species their leaf economics score was either higher or lower compared to their climate score. The best-fit PGLS models showed that MAT and temperature seasonality (Tseason) were significant predictors of SLA (Supplementary Information—Table S2; Fig 1F), while MAT and *T*_max_ emerged as significant predictors of leaf lifespan, and *T*_season_ was a significant predictor of C:N.

### Leaf anatomy and gas exchange versus leaf economics

**Figure 2.**
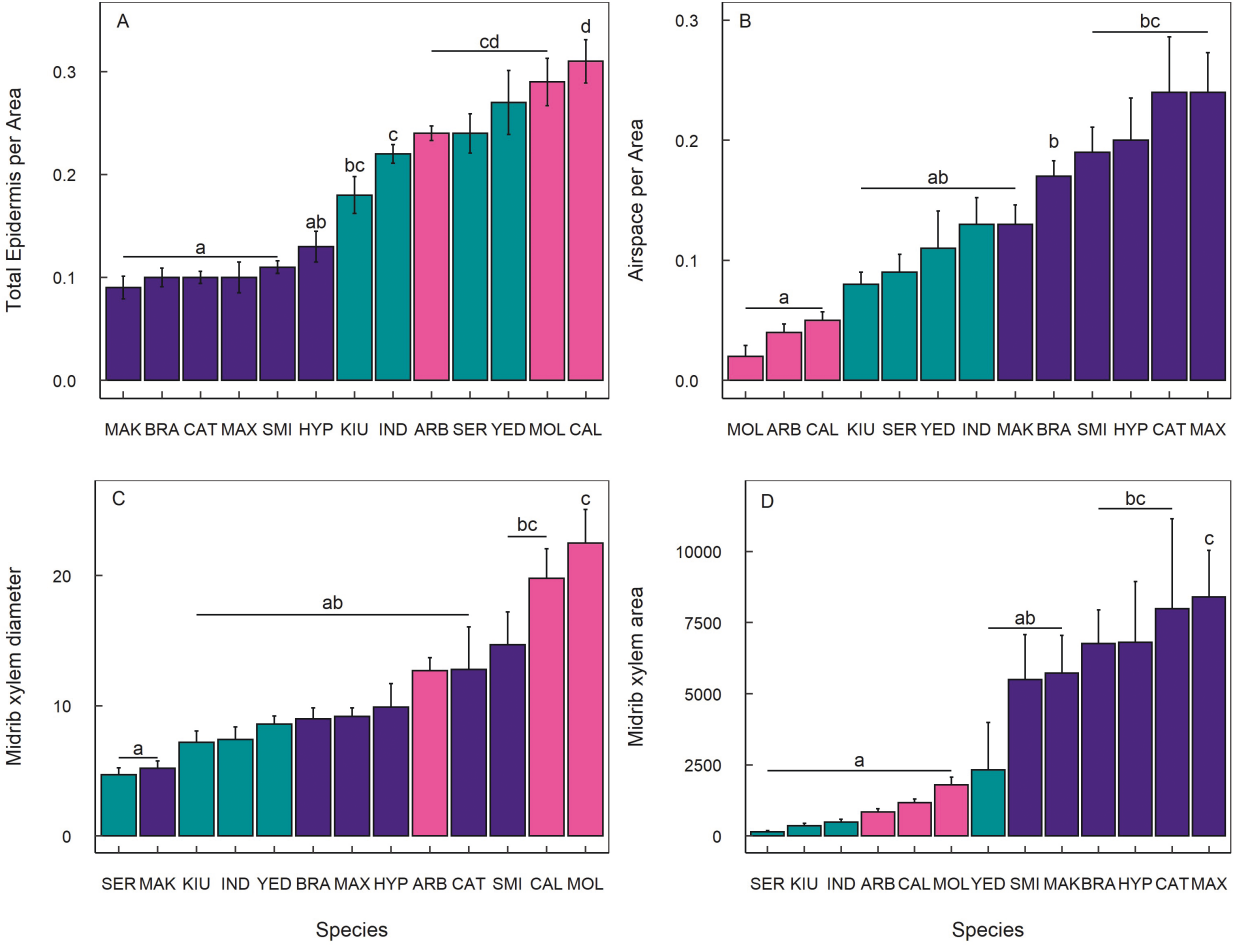
Leaf anatomical properties for 13 *Rhododendron* species representing deciduous, evergreen and semi-evergreen leaf habits, including (a) total epidermis area per leaf area (μm^2^/μm^2^), (b) airspace per leaf area (μm^2^/μm^2^), (c) midrib xylem conduit diameter (μm) and (d) total midrib xylem area (μm^2^). Letter show results of pairwise comparisons, species with different superscript letters are significantly different from each other. Error bars represent standard error. Colours and three-letter species codes are the same as shown in Fig. 1.

We detect a significant effect of C:N on midrib xylem conduit diameter (Table 2A), such that species with higher C:N had smaller conduits (Supplementary Information—Fig. S1), however, leaf anatomy was primarily structured according to leaf habit. Epidermis proportion was significantly higher for deciduous species compared to evergreens, while semi-evergreens showed intermediate values and were not significantly different from either deciduous or evergreen species (Fig. 2A). Airspace proportion was significantly greater for evergreens compared to deciduous species, while semi-evergreens again showed intermediate values and were not significantly different from either deciduous or evergreen species (Fig. 2B). Midrib xylem conduit diameter was significantly larger for deciduous species compared to semi-evergreens, while evergreens displayed intermediate values and were not significantly different from the other leaf habits (Fig. 2C). Midrib xylem area was significantly larger than either deciduous or semi-evergreens, while both deciduous and semi-evergreen species had much lower values and were not significantly different from each other (Fig. 2D).

**Table 2.** *T*-values from PGLS models testing for a relationship of (A) leaf economics traits to leaf anatomical proportions, and (B) leaf traits (leaf economics and leaf anatomy) to leaf gas exchange for 13 *Rhododendron* species representing deciduous, evergreen, and semi-evergreen leaf habits. Bold indicates significance.

A)	SLA	CN	Habit EV	Habit SEV
Epidermis proportion	0.64	−0.25	−2.62*	−1.19
Palisade mesophyll proportion	−1.11	1.45	−1.38	−1.44
Air space proportion	−0.12	−0.84	2.83*	1.90
Midrib xylem conduit diameter	−0.92	−2.77*	−1.65	−4.30**
Total midrib xylem area	0.12	−1.04	3.49**	−0.17

B)	A	E	*g*_s_	WUE
SLA	5.35*	0.12	1.12	1.11
+CN	−3.45*	−5.61*	−3.51*	1.13
+Epidermis†	−5.96**	−3.26*	−2.39	0.46
+Palisade mesophyll†	9.67**	−1.35	0.57	3.41*
+Airspace†	0.23	−5.35*	−4.00*	2.46
+Midrib xylem conduit diameter	−5.36*	0.10	−0.72	−2.48
+Midrib xylem area	−2.54	2.17	1.14	−1.46
+Leaf habit EV	2.62	3.20*	3.48*	−0.80
+Leaf habit SEV	1.12	6.53**	4.47*	−3.36*

*P < 0.05, **P < 0.01.

**Figure 3.**
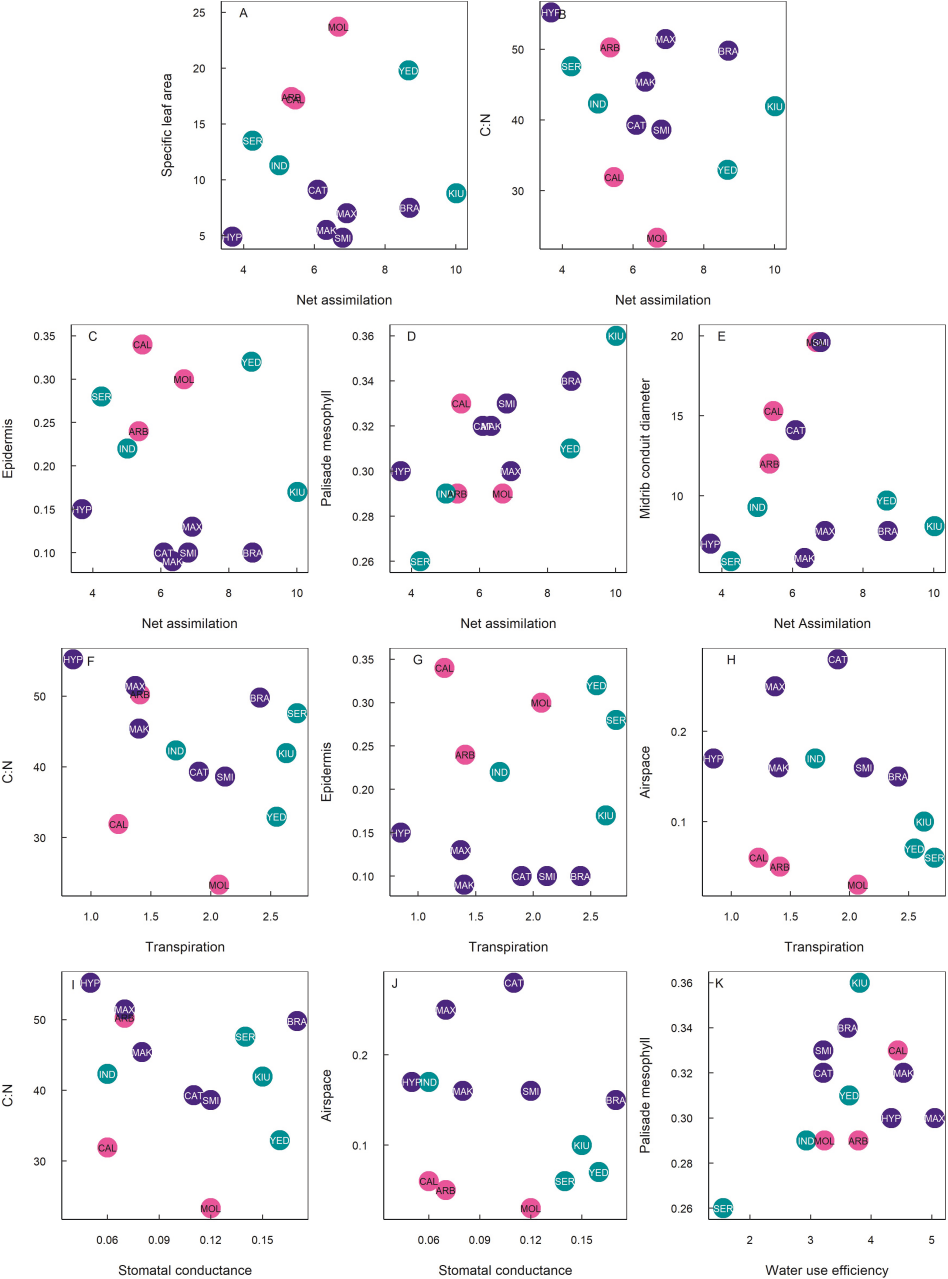
Relationships of leaf gas exchange to leaf economics and leaf anatomy for 13 *Rhododendron* species representing deciduous, evergreen, and semi-evergreen leaf habits, including net assimilation versus (a) specific leaf area, (b) C:N, (c) epidermal proportion, (d) palisade proportion, and (e) midrib conduit diameter; stomatal conductance versus (f) C:N, (g) epidermal proportion, and (h) airspace proportion; transpiration (*E*) versus (i) C:N, and (j) airspace proportion, and (k) water use efficiency versus palisade proportion. Colours and three-letter species codes are the same as shown in Fig. 1. Units are as follows: airspace proportion (μm^2^/μm^2^), C:N (mg/mg), epidermal proportion (μm^2^/μm^2^), midrib conduit diameter (μm), net assimilation (μmol m^−2^ s^−1^); palisade proportion (μm^2^/μm^2^), specific leaf area (m^2^ kg^−1^), stomatal conductance (mmol m^−2^ s^−1^), transpiration (mmol m^−2^ s^−1^), water use efficiency (μmol/mmol).

Net Assimilation (*A*) showed a significant positive relationship to SLA (Table 2B, Fig. 3A) and a significant negative relationship to C:N (Fig. 3B), such that species with a more acquisitive leaf economics traits had higher *A*. We also found that net assimilation was significantly higher for species with a lower epidermis proportion (Fig. 3C), a higher palisade mesophyll proportion (Fig. 3D), and larger midrib xylem conduit diameter (Fig. 3E).

Transpiration (*E*) also showed a significant negative relationship to C:N (Table 2B, Fig. 3F), whereby species with more acquisitive leaf economics had higher transpiration rates. We also found that *E* was significantly greater for species with lower epidermis proportion (Fig. 3G) and lower airspace proportion (Fig. 3). In addition, the relationships of *E* to leaf anatomical traits were structured according to leaf habit (Table 2), with evergreen, semi-evergreen and deciduous species all showing different slopes for the relationships of *E* to C:N, epidermis proportion, and airspace proportion.

Stomatal conductance (*g*_s_) showed a similar pattern of significance as observed for E (Table 2B), being significantly higher for species with lower C:N (Fig. 3I) and lower airspace proportion (Fig. 3J), and being significantly different according to leaf habit, such that deciduous, evergreen and semi-evergreens showed different slopes for the relationships of *g*_s_ to C:N and airspace proportion.

Water use efficiency (WUE) was significantly higher for species with higher palisade mesophyll proportion (Table 2B, Fig. 3K), but this relationship was also influenced by leaf habit, with semi-evergreens showing a significantly steeper positive slope compared to deciduous species, and evergreens showing a negative slope, although this was not significantly different from deciduous species.

### Leaf anatomy versus species climate of origin and other species traits

**Figure 4.**
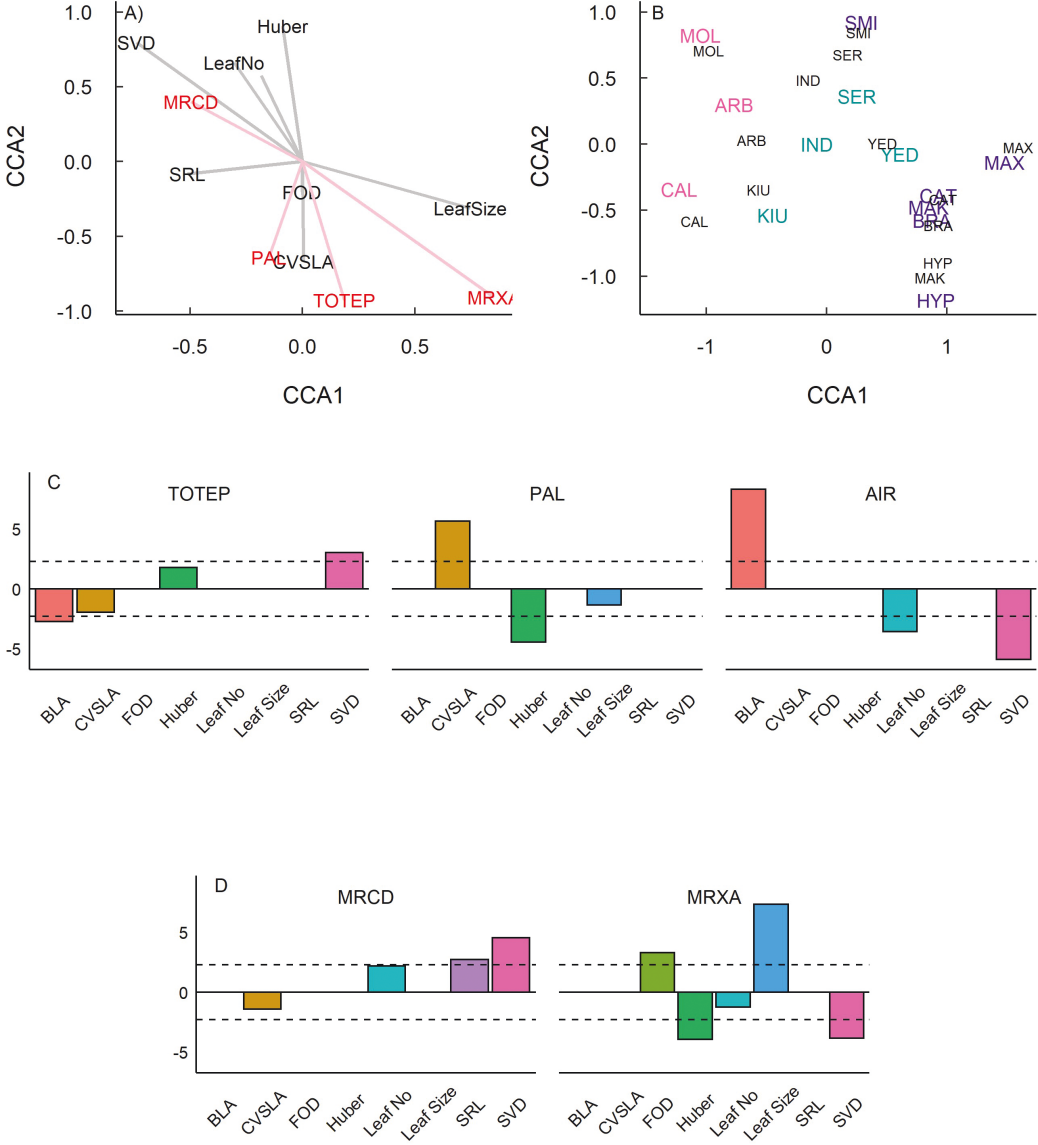
Canonical correspondence analysis (CCA) loadings, species scores and *t*-values from best-fit PGLS models investigating the relationships of leaf anatomy to other species traits for 13 *Rhododendron* species representing deciduous, evergreen, and semi-evergreen leaf habits, including (a) CCA loadings, (b) species CCA scores, and best-fit PGLS models for (c) leaf blade traits and (d) leaf vein traits. For CCA loadings, red indicates leaf anatomical traits, grey indicates other species traits. For CCA scores, colours and three-letter species codes are the same as shown in Fig. 1.

Our CCA did not detect a significant relationship between leaf anatomy and species climate of origin (Table 1). We did detect a significant relationship of leaf anatomy to other species traits along the first canonical axis (CCA1, Table 1). For leaf anatomy, this axis was primarily composed by total midrib xylem area (MRXA) in opposition to midrib conduit diameter (MRCD; Fig. 4A), while CCA2 was characterized by epidermal proportion (TOTEP) and palisade proportion (PAL), with smaller contributions by MRCD and MRXA. For other species traits, the primary components of CCA1 were leaf size in opposition to specific root length (SRL) and a smaller contribution from stem vessel diameter (SVD), while CCA2 was characterized by Huber value, leaf number and branch leaf area (BLA) in opposition to the co-efficient of variation in SLA (CV-SLA) and first order root diameter (FOD). Species scores for both leaf anatomy and other species traits were structured according to leaf habit along CCA1 (Fig. 4B), and across CCA2 four evergreens and one semi-evergreen species showed high correspondence between scores for leaf anatomy and other species traits (BRA, CAT, SMI, MAX, and YED). Best fit PGLS models showed that BLA and SVD were significantly related to TOTEP, while CVSLA and Huber value were significant predictors of PAL, and airspace proportion was significantly related to BLA, leaf number and SVD (Supplementary Information—Table S3; Fig. 4C). For vascular traits, best fit models showed that MRCD was significantly related to SRL and SVD, while MRXA was related to FOD, Huber value, leaf size and SVD (Supplementary Information—Table S3; Fig. 4D).

### Gas exchange versus species climate of origin and other species traits

**Figure 5.**
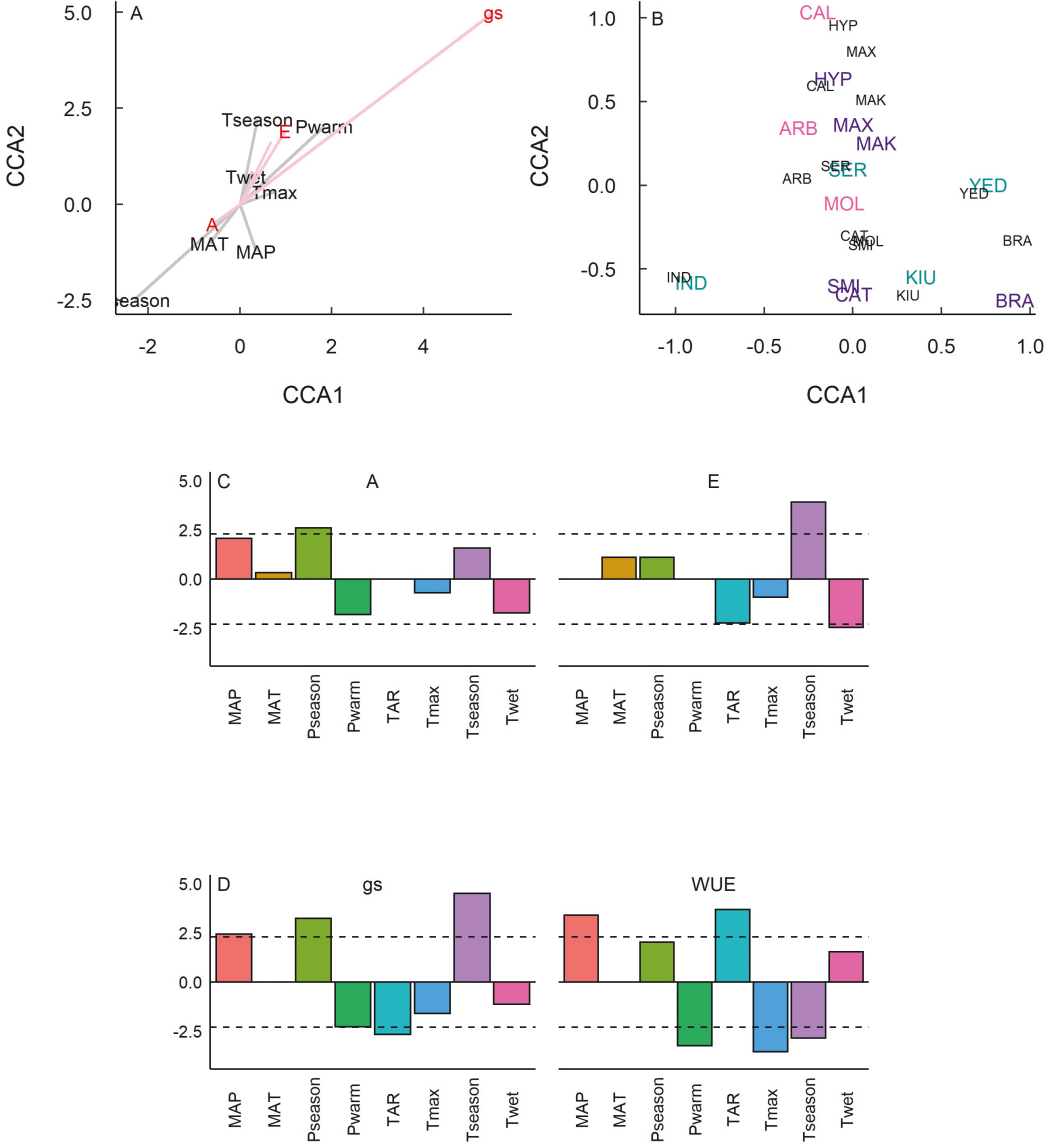
Canonical correspondence analysis (CCA) loadings, species scores and *t*-values from best-fit PGLS models investigating the relationships of leaf gas exchange to species climate of origin for 13 *Rhododendron* species representing deciduous, evergreen and semi-evergreen leaf, including (a) CCA loadings, (b) species CCA scores, and best-fit PGLS models for (c) net assimilation (A) and transpiration (E), along with (d) stomatal conductance (*g*_s_) and water use efficiency (WUE). For CCA loadings, red indicates leaf gas exchange traits, grey indicates climate of origin. For CCA scores, colours and three-letter species codes are the same as shown in Fig. 1.

Our CCA found a significant relationship between gas exchange and species climate of origin along CCA1 (Table 1). For gas exchange, CCA1 was composed primarily by *g*_s_ in opposition to *A*, with smaller contribution by *E*, while CCA2 was composed primarily by *E* and WUE (Fig. 5A). For species climate of origin, CCA1 was composed primarily by *P*_season_ (precipitation seasonality), MAT and MAP (mean annual precipitation) in opposition to *T*_season_ and *P*_warm_ (precipitation in the warmest quarter), while all other climate variables contributed primarily to CCA2 and showed little contribution to overall variance in the data. Species scores along CCA1 showed three major groups, without regard to leaf habit (Fig. 5B), with all deciduous species and all but one evergreen species (BRA) occupying the same central region of the graph, while semi-evergreens were distributed across the full range of CCA1. Semi-evergreens also showed high correspondence between gas exchange and species climate of origin along CCA2, while those for deciduous and evergreen species showed low correspondence along this axis. Our best fit PGLS models found that *P*_season_ was a significant predictor of *A*, while TAR (temperature annual range), *T*_season_ and *T*_wet_ were significant predictors of *E* (Supplementary Information—Table S4; Fig 5C). For *g*_s_, significant predictors included MAP, *P*_season_, *P*_warm_, TAR and *T*_season_, while for WUE significant predictors included MAP, *P*_warm_, TAR, *T*_max_ and *T*_season_ (Supplementary Information—Table S4; Fig. 5D).

Our CCA did not detect a significant relationship between leaf gas exchange and other species traits (Table 1).

## Discussion

While variation in plant traits is often viewed as reflecting adaptation to the environment, the evidence for trait–climate relationships is weak overall, and this lack of relationship may be explained by poor contextualization of individual traits within their evolutionary and whole-plant contexts (Anderegg 2023). Confounding variables may include ancestral traits, which may constrain potential trait combinations within lineages, so comparisons of closely relates species are needed to understand how evolutionary history may shape relationships of individual traits to environment. In addition, examination of traits which specifically integrate function across the whole-plant can contextualize traits to tease apart complex adaptations to environment. Leaf habit has sometimes been dismissed as a simple categorical expression of leaf economics, but the ability of plants to determine the timing of physiological activity may act independently from carbon allocation to create unexpected trait–trait or trait–climate patterns. Similarly, plant architecture (i.e. the size, number and arrangement of plant parts) along with flexibility of that architecture will determine the ratio of supply to demand across plant organs in any given climate. While plant architecture has been well-studied as an integrator of whole-plant function in plant water transport, thus far few studies have included architectural features as a component of whole-plant carbon economics.

We investigated relationships of leaf anatomy and leaf gas exchange to leaf economics and species climate of origin as well as other root, wood and architectural traits for *Rhododendron* species with deciduous, evergreen and semi-evergreen leaf habits. Our results show that species with higher specific leaf area (SLA) do have shorter leaf lifespans as expected based on leaf economics theory (Reich 2014), indicating that the leaf economics spectrum is expressed within genus *Rhododendron*. Our work expands on previous research by showing this pattern holds within a group of closely related species, while controlling for phylogenetic relationships. In addition, we provide a fresh perspective on leaf economics by presenting evidence that SLA and carbon to nitrogen ratio (C:N) represent different dimensions of leaf resource use and acquisition in *Rhododendron*, and that different aspects of leaf economics and leaf anatomy reflect carbon uptake versus water use. Interestingly, our analysis showed that species varied in the strength of correspondence of leaf economics and leaf anatomy with other species traits, where species from the most seasonal climates had the highest correspondence of leaf economics and gas exchange traits with climate scores, suggesting that the strength of trait-trait correlations has both evolutionary and ecological relevance. Below we discuss evidence from our study supporting the idea that leaf habit and plant architecture represent critical aspects which integrate whole-plant carbon and water relations, with implications for plant climate associations of *Rhododendron*.

Intriguingly, we saw a relationship between SLA and net assimilation (*A*) that was structured according to leaf habit, but C:N was related to transpiration (*E*) and stomatal conductance (*g*_s_) in a similar manner across all leaf habits. We saw that species with lower C:N employ an acquisitive whole-plant strategy, having higher gas exchange rates, larger midrib xylem conduits, smaller first order root diameter and lower specific root length. In a previous study that included only SLA we concluded that leaf and root economics were functionally decoupled in *Rhododendron* (Medeiros *et al.* 2017), and this work expands on that by showing that studies that only include SLA may be missing important dimensions of whole-plant economics. Our prior work also showed that C:N is a better predictor of *Rhododendron* stem xylem properties than SLA (Medeiros *et al.* 2020), and this current study adds fascinating evidence for the importance of C:N in plant water relations. Future work should consider the mechanism by which C:N impacts *Rhododendron* water relations, and the possibility that nitrogen limitation may place constraints on stomatal opening and thereby alter carbon allocation. In addition, by examining the relationship of leaf economics to plant architecture our study expanded on previous work by showing that leaf lifespan covaried with total branch leaf area, and deciduous species with the highest SLA had the smallest branch leaf area, suggesting that being acquisitive at the whole-plant level can mean maintaining as little leaf surface area as possible. Among *Viburnum* species leaf lifespan was strongly correlated with branching patterns, and particularly so for branches with terminal inflorescences leaf lifespan was closely related to plant flowering time (Edwards *et al.* 2014), highlighting plant architecture as an integrator of whole-plant function.

Our comparison of leaf anatomy and gas exchange illuminates new information on the mechanisms by which carbon, nitrogen and water relations intertwine to affect whole-plant resource acquisition in *Rhododendron*. In *R. delavayi* mesophyll conductance, not stomatal conductance, sets limits on photosynthesis (Cai *et al.* 2015) and our study adds strength to this conclusion by showing that this holds true across multiple species with different leaf habits, likely due to greater numbers of chloroplasts as mesophyll proportion increases (Deng *et al.* 2012; Cai *et al.* 2014; Gotoh *et al.* 2018). Further work should investigate whether photosynthetic capacity, cell wall properties or stomatal traits drive this pattern (Franks *et al.* 2009). We also found that *E* and *A* were lower for species with a greater proportion of epidermal tissue which may be driven by the fact that the adaxial epidermal layers can prevent sunlight from reaching the palisade mesophyll (Lin and Ehleringer 1983). A larger airspace also resulted in reduced *E* and *g*_s_, but not *A* or water use efficiency, indicating that larger airspace could allow for maintenance of carbon gain during partial stomatal closure. Interestingly, the proportion of airspace and SLA were most strongly structured by leaf habit, and the strength and sometimes even the direction of relationships of *E* and *g*_s_ to leaf economics differed according to leaf habit, suggesting it has emergent effects on the integration of leaf water use and carbon gain of *Rhododendron*.

The relative contributions of leaf anatomy and SLA to gas exchange function can differ according to leaf type (Castro-Díez *et al.* 2000; Buckley *et al.* 2015), and compared to evergreens, deciduous species should have higher gas exchange rates (Givnish 2002; Tomlinson *et al.* 2013). In fact, all *Rhododendron* have rather small xylem conduits which are expected to limit their stomatal conductance under conditions of high evaporative demand (Medeiros *et al.* 2020). In our study both the leaves and stems of deciduous species had more acquisitive xylem anatomy compared to evergreens, which should promote faster rates of water transport for deciduous leaves, but also render them more vulnerable to embolism when high evaporative demand is high (Scoffoni *et al.* 2016). In contrast, when evaporative demand is low, evaporation occurs primarily through the epidermis rather than through stomata (Buckley *et al.* 2017), and we found that deciduous species had larger proportion of leaf invested in epidermal tissue. This suggests that cuticular transpiration should be less of a problem in deciduous leaves, which is consistent with previous studies in *Rhododendron* showing that deciduous leaves allocate more energy to water saving structures (Wang *et al.* 2008).

Compared to the evergreen species examined here, deciduous *Rhododendron* we examined tend to grow in locations that experience hotter summer temperatures and late summer droughts combined with higher light levels (Cox and Cox 1997). High light is associated with smaller leaves and proportionately smaller palisade mesophyll (Deng *et al.* 2012) combined with thicker epidermis (Terashima *et al.* 2011), which is consistent with our findings here for deciduous versus evergreen *Rhododendron*. Previous work has shown that light levels could be a factor driving leaf trait differences across *Rhododendron* (Wang *et al.* 2020), and our data showing that leaf anatomy was clearly related to plant architecture also points to light environment as a key feature shaping their whole-plant coordination. It is important to note here that the plant architecture of *Rhododendron* reduces self-shading, including whorled leaf arrangement and leaves concentrated at the tips of branches. Thus, for *Rhododendron* spatial variation in light availability is most likely to arise from differences in leaf aspect and the composition of the overstory, rather than changes in self-shading as leaves age. While lower light levels can be associated with higher SLA and larger leaves (Wang *et al.* 2020), evergreen *Rhododendron* have lower SLA combined with larger leaves. Plants exposed to lower light levels may also have lower midrib hydraulic conductance (Dunbar‐Co *et al.* 2009), and we found that evergreen species had smaller xylem conduits in both their leaves and wood. This was, however, combined with a larger total midrib xylem area for evergreens, which could buffer the impact of small conduits for shade tolerant leaves (Cavender-Bares *et al.* 2005; Poorter *et al.* 2010; Choat *et al.* 2011). Given the low SLA and longer leaf lifespan of the evergreen species we examined here (3–6 years, Cox and Cox 1997), smaller vessels in the stem and leaf could ensure slow but steady water distribution across the entire canopy (Reinhardt *et al.* 2019), and support photosynthetic productivity by reducing the incidence of embolism (Scoffoni *et al.* 2016). Our finding of higher proportion of airspace for evergreen leaves could be also beneficial for gas exchange in low light (Tomás *et al.* 2013; Earles *et al.* 2018). While plants exposed to lower light levels may have thinner mesophyll (Dunbar‐Co *et al.* 2009), lower SLA can be associated with higher photosynthetic rates, owing to counter-intuitive changes in the thickness of the mesophyll (Edwards *et al.* 2014).

The use of species growing together in botanical gardens for our study provides support for the idea that genetic differences underlie the patterns we observed because the studied plants all grew under the same climate conditions (Primack *et al.* 2021). Botanical gardens typically plant species in their preferred light conditions, however, i.e. species from sunny climates are planted in sunny locations, and species from shade-prone habitats are planted beneath shady overstory, and this was true of the plants examined here. Thus, our design lacks the power to fully disentangle the relative contribution of genetic versus environmental factors to species differences. Previous work using the same botanical garden collections studied here have shown strong variation in SLA along the branch for species in Section Ponticum and Tsutsusi (Medeiros *et al.* 2020), and future work should investigate the role of leaf plasticity in determining the functional attributes of *Rhododendron* leaf traits and phenology in relation to light environments (Primack *et al.* 2021).

In addition to responding to light conditions, leaf anatomical proportions can change across environmental gradients (Wang *et al.* 2008; Shen *et al.* 2017; Zhou *et al.* 2017), but we did not observe any relationship between leaf anatomy and climate variables examined in this study. Instead, we found evidence that *Rhododendron* originating from climates with higher mean annual temperature and higher temperature seasonality had the highest SLA, shortest leaf lifespan and lowest C:N. Plants from more stressful climates conditions often have lower SLA (Poorter *et al.* 2009), but the components of SLA can change independently across growth environments (Sancho-Knapik *et al.* 2021), and there may be differences in the magnitude of trait variation across organs within a plant, leading to unexpected relationships (Medeiros *et al.* 2020). Our investigation of semi-evergreen species sheds additional light on the role for suites of traits in negotiating highly seasonal environments. Semi-evergreen *Rhododendron* tend to have higher SLA and occupy drier environments with higher levels of irradiance compared with evergreen species (Cai *et al.* 2014). In contrast we found that semi-evergreens had the smallest midrib conduit diameter and lowest midrib xylem area, suggesting that they should show the most conservative water use. Instead, semi-evergreens had the highest leaf water use and lowest water use efficiency but this was combined with high Huber value driven by incredibly tiny leaves (Royal Botanic Garden Edinburgh. 2010). This current study expands on this previous work by showing that leaf economics, leaf habit and plant architecture work together to define the carbon uptake and water relations of *Rhododendron* originating from different climates. Semi-evergreens also have high variation in SLA along the branch (i.e. CVSLA; Medeiros *et al.* 2020), suggesting they implement a more acquisitive approach to water use efficiency by producing a leaf canopy that is exceptionally cheap in terms of carbon and flexible in terms of the size of the canopy and the timing of phenology. Interpretation of our results must also, however, consider that the relationships between traits and climate may differ according to plant growing conditions (Ramírez-Valiente *et al.* 2020), so additional studies will be needed which examine how trait co-variation could be altered under different environment contexts.

## Supporting Information

The following additional information is available in the online version of this article –

Figure S1: Phylogenetic least squares showed that species with higher C:N had significantly smaller leaf midrib xylem conduit diameter. Colours indicate leaf habits and three letter codes indicate species names, purple = evergreen species (BRA, *R. brachycarpum*, CAT, *R. catawbiense*, HYP, *R. hyperythrum*, MAK, *R. makinoi*; MAX, *R. maximum*; SMI, *R. smirnowii*), teal = semi-evergreen species (IND, *R. indicum*; KIU, *R. kiusianum*; SER, *R. serpyllifolium*; YED, *R. yedoense*), pink = deciduous species (ARB, *R. arborescens*; CAL, *R. calendulaceum*; MOL, *R. molle*).

Table S1: Model selection determining best-fit model for leaf economics as a function of species traits. Bold indicates selected model.

Table S2. Best-fit models for relationship of leaf economics to climate. Bold indicates selected model. SLA = specific leaf area; lifespan = leaf lifespan; CN = leaf carbon to nitrogen ratio.

Table S3. Best-fit models for relationship of leaf anatomical traits to species traits. Bold indicates selected model. Anatomical variable codes are as follows: total epidermis area (TOTEP), proportion of palisade mesophyll (PAL), proportion of airspace (AIR), mean midrib conduit diameter (MRCD), and total midrib xylem area (MRXA).

Table S4. Best-fit model for relationship of gas exchange traits to climate. Bold indicates selected model.

plae005_suppl_Supplementary_Material

## Data Availability

The data and code underlying this article are available in GitHub, at https://github.com/JulianaSMedeiros/Medeiros-et-al-leaf-anatomy-synthesis
