# Supplementary material for: Leaf habit and plant architecture integrate whole-plant economics and contextualize trait–climate associations within ecologically diverse genus Rhododendron
Source: AoB Plants. 2024 Feb 6;16(2):plae005. doi: 10.1093/aobpla/plae005 (PMC10888519; doi:10.1093/aobpla/plae005)
Supplement: plae005_suppl_Supplementary_Material [file plae005_suppl_supplementary_material.pdf]

Leaf habit and plant architecture integrate whole-plant economics and contextualize trait-climate associations within ecologically diverse genus *Rhododendron*

Juliana S. Medeiros, Jean H. Burns, Callie Dowrey, Fiona Duong, and Sarah Speroff

Supporting information

Figure S1: Phylogenetic least squares showed that species with higher C:N had significantly smaller leaf midrib xylem conduit diameter. Colors indicate leaf habits and three letter codes indicate species names, purple = evergreen species (BRA, *R. brachycarpum*, CAT, *R. catawbiense*, HYP, *R. hyperythrum*, MAK, *R. makinoi*; MAX, *R. maximum*; SMI, *R. smirnowii*), teal = semi-evergreen species (IND, *R. indicum*; KIU, *R. kiusianum*; SER, *R. serpyllifolium*; YED, *R. yedoense*), pink = deciduous species (ARB, *R. arborescens*; CAL, *R. calendulaceum*; MOL, *R. molle*).

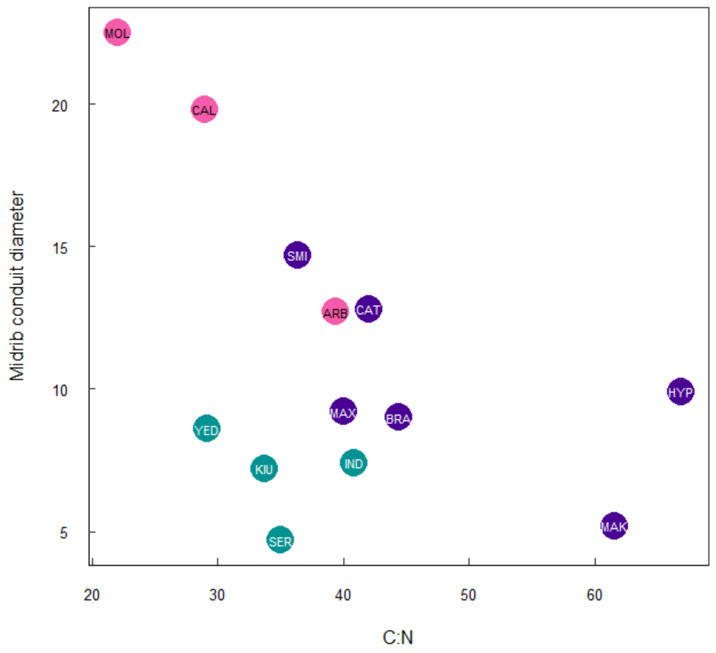

Table S1: Model selection determining best-fit model for leaf economics as a function of species traits. Bold indicates selected model.

Variable	SRL	FOD	Stem Vessel Diameter	CVSLA	Leaf Size	Leaf No	Branch Leaf Area	Huber	Adj. R2
SLA	1.72	0.67	4.04*	-2.99*	0.68	1.63	-1.30	4.20*	0.879
SLA	1.72	-	4.24**	-3.39*	0.50	1.58	-1.20	4.47**	0.892
**SLA**	**2.31**	**-**	**5.09****	**-3.72****	**-**	**1.81**	**-2.34**	**5.05****	**0.905**
SLA	1.51	-	4.78**	-2.88*	-	-	-1.64	4.34**	0.875
SLA	-	-	4.74**	-3.50**	-	-	-1.07	4.39**	0.854
SLA	-	-	6.15***	-3.60**	-	-	-	5.37***	0.852
SLA	-	-	4.59***	-	-	-	-	2.81*	0.691
SLA	-	-	3.54**	-	-	-	-	-	0.490
Lifespan	-1.65	1.33	-1.18	-0.15	0.05	1.14	1.40	-0.59	0.940
Lifespan	-1.87	1.50	-1.65	-0.16	-	1.86	6.18**	-0.66	0.952
**Lifespan**	**-2.04**	**1.71**	**-1.97**	**-**	**-**	**2.13**	**7.25*****	**-1.33**	**0.960**
Lifespan	-2.27	1.17	-1.39	-	-	3.13*	8.85***	-	0.955
Lifespan	-5.16***	-	-1.13	-	-	3.09*	9.10***	-	0.953
Lifespan	-4.97***	-	-	-	-	5.74***	8.90***	-	0.951
Lifespan	-	-	-	-	-	4.55**	3.96**	-	0.855
Lifespan	-	-	-	-	-	4.94***	-	-	0.661
CN	2.05	3.64*	-2.65	-0.75	1.15	0.86	-1.77	-1.14	0.788
CN	2.03	4.11**	-4.02*	-	0.93	0.47	-1.86	-3.48*	0.806
CN	2.15	4.45**	-6.71***	-	0.87	-	-1.97	-4.09**	0.831
**CN**	**2.81***	**4.44****	**-6.80*****	**-**	**-**	**-**	**-2.67***	**-4.12****	**0.837**
CN	1.50	3.02*	-4.82**	-	-	-	-	-2.43*	0.712
CN	-	3.53*	-6.20***	-	-	-	-	-2.02	0.766
CN	-	2.82*	-5.61***	-	-	-	-	-	0.712
CN	-	-	-3.46**	-	-	-	-	-	0.478

*P<0.05, **P<0.01, ***P<0.001

Table S2. Best-fit models for relationship of leaf economics to climate. Bold indicates selected model. SLA = specific leaf area; lifespan = leaf lifespan; CN = leaf carbon to nitrogen ratio

Variable	MAT	MAP	Tseason	Pseason	Tmax	TAR	Pwarm	Twet	Adj. R2
SLA	1.85	0.01	1.39	0.27	-1.40	0.90	-0.21	-0.51	0.574
SLA	2.12	-	1.58	0.33	-1.58	1.01	-0.56	-0.68	0.659
SLA	2.41	-	2.16	-	-1.82	1.04	-0.52	-0.71	0.710
SLA	2.73*	-	2.22	-	-2.02	0.97	-	-1.19	0.740
**SLA**	**2.68***	**-**	**2.70***	**-**	**-1.87**	**-**	**-**	**-1.39**	**0.742**
SLA	2.53*	-	2.43*	-	-1.13	-	-	-	0.698
SLA	4.96***	-	3.09*	-	-	-	-	-	0.672
SLA	2.90*	-	-	-	-	-	-	-	0.381
Lifespan	-1.91	0.72	-1.39	0.27	1.56	-0.82	-0.80	1.06	0.334
Lifespan	-2.12	0.75	-1.61	-	1.70	-1.00	-0.85	1.14	0.457
Lifespan	-2.13	-	-1.72	-	1.74	-1.02	-0.42	0.92	0.498
**Lifespan**	**-3.08***	**-**	**-2.06**	**-**	**2.73***	**-1.22**	**-**	**0.87**	**0.557**
Lifespan	-2.98*	-	-2.15	-	2.40*	-1.30	-	-	0.505
Lifespan	-2.95*	-	-2.52*	-	2.29*	-	-	-	0.426
Lifespan	-1.51	-	-0.81	-	-	-	-	-	0.434
Lifespan	-1.21	-	-	-	-	-	-	-	0.037
CN	-1.18	1.04	-0.88	0.62	1.04	-0.31	-0.79	0.66	-0.005
CN	-1.25	1.19	-1.26	0.74	1.09	-	-0.91	0.92	0.176
CN	-1.09	0.98	-1.15	-	0.93	-	-0.56	0.78	0.239
CN	-1.35	1.36	-1.62	-	1.29	-	-	0.61	0.313
CN	-1.42	1.48	-1.94	-	1.27	-	-	-	0.367
CN	-0.86	0.77	-2.02	-	-	-	-	-	0.325
CN	-0.71	-	-2.85*	-	-	-	-	-	0.352
**CN**	**-**	**-**	**-2.90***	**-**	**-**	**-**	**-**	**-**	**0.381**

*P<0.05, **P<0.01, ***P<0.001

Table S3. Best-fit models for relationship of leaf anatomical traits to species traits. Bold indicates selected model. Anatomical variable codes are as follows: total epidermis area (TOTEP), proportion of palisade mesophyll (PAL), proportion of airspace (AIR), mean midrib conduit diameter (MRCD), and total midrib xylem area (MRXA)

Variable	SRL	FOD	Stem Vessel Diameter	CVSLA	Leaf Size	Leaf No	Branch Leaf Area	Huber	Adj. R2
TOTEP	0.31	-0.20	1.65	-1.01	-0.17	0.42	-0.51	1.22	0.648
TOTEP	0.32	-0.16	2.42	-1.42	-	0.88	-2.38	1.68	0.716
TOTEP	0.62	-	2.66*	-1.89	-	1.02	-2.66*	1.91	0.762
TOTEP	-	-	2.71*	-2.01	-	0.88	-2.79*	1.94	0.783
**TOTEP**	**-**	**-**	**3.04***	**-1.96**	**-**	**-**	**-2.72***	**1.81**	**0.789**
TOTEP	-	-	2.48*	-0.89	-	-	-3.50**	-	0.736
TOTEP	-	-	2.81*	-	-	-	-3.43**	-	0.741
TOTEP	-	-	-	-	-	-	-4.18**	-	0.579
PAL	1.59	0.85	-1.15	1.91	-0.39	-0.69	-0.13	-2.62	0.339
PAL	1.78	0.97	-1.60	2.55	-1.81	-1.35	-	-3.32*	0.470
PAL	1.51	-	-1.67	3.48*	-1.80	-1.56	-	-3.62*	0.475
PAL	-	-	-1.33	3.92**	-1.66	-1.18	-	-3.50*	0.541
PAL	-	-	-0.48	4.57**	-1.37	-	-	-3.94**	0.599
**PAL**	**-**	**-**	**-**	**5.66*****	**-1.33**	**-**	**-**	**-4.45****	**0.711**
PAL	-	-	-	5.34**	-	-	-	-4.13**	0.690
PAL	-	-	-	1.10	-	-	-	-	0.016
AIR	-0.74	-0.01	-2.48	0.77	0.49	-0.99	0.57	-1.00	0.770
AIR	-0.99	-	-2.86*	1.04	0.59	-1.20	0.69	-1.18	0.816
AIR	0.82	-	-4.71**	1.13	-	-3.32*	4.85**	-1.26	0.922
AIR	-	-	-4.84**	1.46	-	-3.07*	5.03**	-1.57	0.895
AIR	-	-	-5.42***	-	-	-3.42**	6.72***	-0.71	0.928
**AIR**	**-**	**-**	**-5.88*****	**-**	**-**	**-3.56****	**8.35*****	**-**	**0.932**
AIR	-	-	-3.24**	-	-	-	4.26**	-	0.809
AIR	-	-	-	-	-	-	4.76***	-	0.643
MRCD	1.72	0.58	2.50	-0.89	-0.09	0.85	0.19	0.40	0.532
MRCD	1.93	0.73	3.53*	-1.21	-	1.50	0.34	0.58	0.624
MRCD	2.49*	0.88	3.81**	-1.34	-	1.84	-	0.58	0.680
MRCD	2.55*	0.78	4.38**	-1.58	-	2.15	-	-	0.711
**MRCD**	**2.75***	**-**	**4.55****	**-1.41**	**-**	**2.19**	**-**	**-**	**0.723**
MRCD	3.41**	-	4.28**	-	-	1.90	-	-	0.694
MRCD	2.63*	-	3.75**	-	-	-	-	-	0.615
MRCD	-	-	3.04*	-	-	-	-	-	0.407
MRXA	-0.29	1.62	-2.10	-0.13	1.79	-0.31	-0.22	-1.48	0.893
MRXA	-0.35	2.10	-3.03*	-	2.22	-0.69	-0.19	-3.32*	0.914
MRXA	-0.36	2.29	-3.57*	-	6.13***	-1.20	-	-3.63*	0.928
**MRXA**	**-**	**3.30***	**-3.83****	**-**	**7.35*****	**-1.23**	**-**	**-3.93****	**0.937**
MRXA	-	2.97*	-4.18**	-	7.22***	-	-	-3.65**	0.933
MRXA	-	-	-3.35**	-	4.83***	-	-	-2.23	0.874
MRXA	-	-	-2.47*	-	5.79***	-	-	-	0.824
MRXA	-	-	-	-	5.96***	-	-	-	0.742

*P<0.05, **P<0.01, ***P<0.001

Table S4. Best-fit model for relationship of gas exchange traits to climate. Bold indicates selected model.

Variable	MAT	MAP	Tseason	Pseason	Tmax	TAR	Pwarm	Twet	Adj. R2
A	0.36	1.87	1.31	2.32	-0.62	0.21	-1.63	-1.43	0.760
**A**	**0.33**	**2.07**	**1.58**	**2.60***	**-0.70**	**-**	**-1.81**	**-1.73**	**0.806**
A	-	1.28	3.51*	2.45*	-1.89	-	1.11	-1.53	0.710
A	-	0.81	3.15*	2.10	-1.25	-	-	-1.72	0.610
A	-	-	3.52*	2.41*	-1.41	-	-	-1.56	0.626
A	-	-	3.06*	3.40**	-	-	-	-2.65*	0.585
A	-	-	2.44*	1.69	-	-	-	-	0.334
A	-	-	2.10	-	-	-	-	-	0.221
E	0.63	0.04	3.14*	0.71	-0.49	-1.82	0.10	-1.66	0.736
E	0.73	-	3.57*	0.85	-0.56	-2.05	0.36	-2.20	0.789
**E**	**1.10**	**-**	**3.91****	**1.10**	**-0.93**	**-2.24**	**-**	**-2.47***	**0.819**
E	0.97	-	3.91**	1.69	-	-3.22*	-	-2.09	0.801
E	-	-	4.10**	1.57	-	-3.09*	-	-1.84	0.783
E	-	-	5.69***	-	-	-4.80***	-	-1.19	0.751
E	-	-	5.71***	-	-	-4.74***	-	-	0.728
E	-	-	2.10	-	-	-	-	-	0.222
gs	0.53	2.08	3.60*	2.75	-0.77	-1.78	-2.11	-1.13	0.896
**gs**	**-**	**2.43**	**4.50****	**3.24***	**-1.60**	**-2.67***	**-2.28**	**-1.14**	**0.911**
gs	-	2.44	3.99**	3.14*	-3.52*	-2.28	-2.84*	-	0.871
gs	-	1.13	2.85*	2.84*	-3.11*	-	1.45	-	0.713
gs	-	-	2.99*	2.95*	-2.85*	-	-1.41	-	0.702
gs	-	-	4.06**	2.96*	-2.73*	-	-	-	0.670
gs	-	-	2.61*	2.56*	-	-	-	-	0.457
gs	-	-	1.92	-	-	-	-	-	0.183
WUE	-0.12	2.99*	-2.09	1.77	-0.44	2.71	-2.91*	1.38	0.610
**WUE**	**-**	**3.39***	**-2.87***	**2.02**	**-3.55***	**3.69***	**-3.25***	**1.54**	**0.687**
WUE	-	2.73*	-2.21	1.66	-3.41*	3.03*	-2.66*	-	0.615
WUE	-	2.02	-1.46	-	-2.86*	2.28	-1.86	-	0.518
WUE	-	2.83*	-	-	-2.33*	0.01	-3.23*	-	0.449
WUE	-	1.40	-	-	-1.09	-	-1.81	-	0.094
WUE	-	1.36	-	-	-	-	-1.41	-	-0.001
WUE	-	-	-	-	-	-	-0.36	-	-0.078

*P<0.05, **P<0.01, ***P<0.001
